# Detection of germ cell neoplasia in situ and testicular cancer risk in men with testicular microlithiasis: Real world results through 10 years

**DOI:** 10.1111/andr.70071

**Published:** 2025-05-21

**Authors:** Karoline Skov Lundager, Rasmus Hassing Frandsen, Emil Durukan, Nadia Zeeberg Belhouche, Christian Fuglesang S. Jensen, Peter Busch Østergren, Jens Sønksen, Mikkel Fode

**Affiliations:** ^1^ Department of Urology Copenhagen University Hospital Herlev and Gentofte Hospital Herlev Denmark; ^2^ Department of Clinical Medicine Faculty of Health and Medical Sciences University of Copenhagen Copenhagen Denmark

**Keywords:** germ cell neoplasms, microlithiasis, semen, testicular biopsy, testicular carcinoma in situ, testicular microcalcification, testicular neoplasms

## Abstract

**Background:**

Guidelines recommend biopsies for men <50 years with testicular microlithiasis and cancer risk factors to rule out germ cell neoplasia in situ. Limited data support this practice.

**Objectives:**

To clarify the significance of testicular microlithiasis by examining pathological findings in men with testicular microlithiasis.

**Materials and methods:**

We reviewed charts of men diagnosed with testicular microlithiasis at a tertiary referral center from 2013 to 2023. Patient characteristics, clinical findings, and cancer risk factors including testicular hypotrophy (volume ≤12 mL), infertility, and cryptorchidism were recorded. Men with unknown fertility were offered semen analyses. Histological findings from testicular biopsies and subsequent cancers were noted. Primary endpoints were rates of germ cell neoplasia in situ and testicular cancer diagnoses.

**Results:**

We included 334 men (median age 33 years, range 16–73 years): 27 had testicular hypotrophy, 18 infertility, 25 cryptorchidism, and 56 multiple risk factors. The remaining 208 men had no apparent risk factors. Of these 36 were had reduced semen quality. Overall, 137 of 334 men (41%) underwent biopsies, with germ cell neoplasia in situ in 10 cases (7.3%, 95% confidence interval 3.6%–13%). Four had multiple risk factors (hypotrophy and infertility in two; hypotrophy, infertility, and cryptorchidism in two), three had hypotrophy alone, one had infertility, and two had reduced semen quality. Germ cell neoplasia in situ was unilateral in all cases and only found in testicles with testicular microlithiasis. Unilateral orchiectomy was performed in all germ cell neoplasia in situ cases, with hypotrophy found in all but one. Over a median follow‐up of 4.7 years (range 1.16–11.49 years), testicular cancer developed in three men (0.9%, 95% confidence interval 0.19%–2.6%).

**Discussion:**

Germ cell neoplasia in situ was only detected in cases with both testicular microlithiasis and testicular hypotrophy, and the rate of subsequent cancer development was low. This suggests that testicular microlithiasis alone does not increase cancer risk in otherwise morphologically normal testicles.

**Conclusion:**

Biopsies should only be considered in men with incidental testicular microlithiasis if the testicular size is reduced.

## 1 INTRODUCTION

Testicular microlithiasis (TM) is defined as one or more intratesticular calcifications, with a diameter <3 mm, typically detected incidentally during scrotal ultrasound (US) examinations. The reported prevalence of TM in asymptomatic men varies between 1.1% and 5.6%.^1^, ^2^, ^3^ Histologically two types of calcifications are described: a rounded concentric laminated calcification and an amorphous calcification of hematoxylin bodies from calcific debris.^4^ However, the genesis is unknown, and no distinction is made in clinical practice. Likewise, a traditional subdivision of TM into a limited type with fewer than five calcifications per testicle, and classic type with five or more calcifications per testicle has been abandoned in the clinic as studies have demonstrated that this subdivision lacks clinical significance.^5^, ^6^

Due to a suspected association between TM and germ cell neoplasia in situ (GCNIS), the current Guidelines of the European Association of Urology recommend testicular biopsies in men <50 years old with bilateral TM without any other risk factors or unilateral TM with at least one risk factor to exclude GCNIS.^6^ The risk factors include male infertility, small testicular size (defined in the European Urological guidelines as a testis volume ≤12 mL^6^), and cryptorchidism. In addition to this, the national Danish Urology guidelines recommend that men with TM and unknown fertility status should be offered semen analysis and bilateral testicular biopsies in case of reduced semen quality.^7^ However, we have recently pointed out that the basis of the recommendation stems from small retrospective series with potential biases in patient selection and conclusions that were not adequately justified by the actual findings.^8^ Furthermore, we presented the largest case series to date on biopsy findings in men with TM and were only able to confirm that men with a testis volume ≤12 mL were at elevated risk of harboring GCNIS.^8^ The situation in men with a history of infertility and/or reduced semen quality was less clear as only 38 biopsies were performed on this indication with identification of two patients with GCNIS. Building on our previous study, which focused solely on men who had undergone biopsies, we aimed to further clarify the clinical significance of TM by examining the clinical trajectory and potential pathological findings in a comprehensive cohort of consecutive men with TM.

## 2 MATERIALS AND METHODS

In this retrospective cohort study, we collected data from all patients diagnosed with TM at Herlev and Gentofte Hospital, Denmark, between January 1, 2013, and January 1, 2023. Data collection took place in June 2024. Of note, this patient group overlaps with that of our group's previous publication on biopsy results in men with TM.^8^ The difference is that the previous study focused exclusively on men who had undergone testicular biopsies.

Data were collected from the electronic health records, where patients were identified through the diagnostic code for TM. The comprehensive nature of the Danish electronic patient chart system enabled complete follow‐up regarding cancer development for all patients throughout the study period even if they moved to a different region.

Infertility and cryptorchidism were assessed based on patient history in the health record, while the testicular size had been appraised by the treating physicians either by US or by physical examination. If no other risk factors were identified, semen analyses were offered to patients with unknown fertility status. Reduced semen quality was defined as ≥1 parameters under the WHO laboratory manual 6th edition's lower limit criteria for the total number of spermatocytes, concentration of spermatocytes, total and progressive motility, and morphology.^9^ Patients with TM and hypotrophic testicles (<12 mL), a history of cryptorchidism, infertility, or reduced semen quality, were offered bilateral testicular biopsies to rule out GCNIS. As a standard, biopsies were performed via a scrotal incision under either local or general anesthesia. A single core sample measuring 3 × 3 × 3 mm was taken as this has previously been validated with a high diagnostic efficacy in detecting GCNIS with a false‐negative rate of 0.5%.^10^ The standard treatment for men with unilateral GCNIS was orchiectomy, while men with bilateral GCNIS were offered orchiectomy and radiotherapy.

We recorded indications for initial US, laterality of TM, and presence of testicular size <12 mL, history of cryptorchidism, or infertility. We also noted the patients’ age at TM diagnosis, any known family history of testicular cancer, and if relevant, the result of semen analyses, biopsy‐related complications, pathological findings, and treatments of GCNIS. Finally, we noted if there were subsequent development of testicular cancer for all the patients. Data were collected and managed using the software platform Research Electronic Data Capture (REDCap).^11^ The primary endpoints were the proportion of men diagnosed with GCNIS and/or testicular cancer.

Descriptive statistics were performed. Numerical data were described by median and ranges, while categorical data were described by counts and percentages. Statistical analyses were conducted using SAS Statistical analyses were conducted using SAS Enterprise Guide Version (Institute Inc.). The study was registered and approved by the Regional Center for Register Research of the Capital Region of Denmark according to Danish law (journal number H‐23073357). Patients would be excluded if they had stated that they did not consent to retrospective chart reviews. The manuscript was prepared according to the STROBE statement (www.strobe‐statement.org).

## 3 RESULTS

During the study period, 337 patients were diagnosed with TM. Two patients were excluded as the initial US scans also showed lesions clearly suspicious for testicular cancer and another was excluded because he had declined to participate in retrospective studies. No other patients with a diagnosis of TM were excluded. The median age of the remaining 334 men was 33 years (range 16–73 years). TM was bilateral in 241 (72.2%) and unilateral in 93 (27.8%). Tables 1 and 2 show the distribution of patients according to the indication for US and risk factors respectively. Nine patients had a documented family history of testicular cancer.

**TABLE 1:** Distribution of patients based on indications for initial scrotal ultrasound (US).

US indication	Number of men (percent)
Other	35 (10.5%)
Suspected cancer	82 (24.6%)
Hydrocele	2 (0.6%)
Infection	8 (2.4%)
Infertility	38 (11.4%)
Pain	141 (42.2%)
Spermatocele	4 (1.2%)
Suspected testicular torsion	19 (5.7%)
Varicocoele	5 (1.5%)

*Note*: Indication “other” included US in relation to inguinal hernia, scrotal swelling, yearly health check, and penile disorders.

**TABLE 2:** Distribution of patients based on clinically identified risk factors.

Risk factor	Prevalence
Hypotrophy	27 (8.1%)
Infertility	18 (5.4%)
History of cryptorchidism	25 (7.5%)
Unknown fertility status	126 (35.3%)
Multiple risk factors	56 (16.8%)
No risk factors	82 (24.6%)

Overall, 137 of 334 men (41%) underwent testicular biopsies with detection of GCNIS in 10 cases (3%; 95% confidence interval [CI] 1.4%–5.4%) corresponding to a specific detection rate in biopsied patients of 7.3% (95% CI 3.6%–13%). Among the patients diagnosed with GCNIS, four had multiple risk factors (two had both testicular hypotrophy and infertility, while two had hypotrophy, infertility, and a history of cryptorchidism). Three patients had only testicular hypotrophy, one had only infertility, and two had reduced semen quality. TM was bilateral in seven of the ten cases and unilateral in three of the ten cases, while GCNIS was unilateral in all cases. All three men with unilateral TM had undergone bilateral biopsies, and GCNIS was found exclusively in testicles with TM. Following subsequent orchiectomy, early‐stage testicular cancer was identified in five of the ten patients. Notably, the pathology report showed testicular hypotrophy in nine out of 10 testicles with GCNIS, with a median size of 3.5 mL (ranging from 2 to 9.5 mL). The remaining GCNIS‐positive testicle measured 12 mL.

During a median follow‐up of 4.7 years (range 1.16–11.49 years) from TM diagnosis, three patients (0.9%, 95% CI 0.19%–2.6%) developed testicular cancer. Two of these men had undergone testicular biopsies without signs of GCNIS because of reduced semen quality and an hypotrophic testicle plus a history of cryptorchidism, respectively. The last patient had not undergone biopsies as he had no additional risk factors. None of the nine patients with familial testicular cancer—four of whom had biopsies—were diagnosed with neither GCNIS or subsequent cancer. Detailed analyses of each group, categorized by risk factors, are presented below.

### 3.1 Hypotrophy

A testicular size <12 mL was noted as the only risk factor in 27 of 334 men (8.1%). Fifteen of these men were offered biopsies and 14 of them accepted. Of note, the biopsies were not offered in two cases as the patients had concomitant Klinefelter syndrome as an explanation for their testicular hypotrophy. GCNIS was detected in three of 14 cases corresponding to 21.4% (95% CI 4.7%–50.8%). On the pathology reports after unilateral orchiectomy early‐stage cancer was present in two of three of the GCNIS carrying testicles.

### 3.2 Infertility

Eighteen of 334 men (5.4%) had a history of male factor infertility as their only risk factor. In these men, 17 were offered biopsies and 15 accepted. GCNIS was detected in one of 15 (6.7%, 95% CI 0.17%–32.0%). On the pathology report, only GCNIS was found, and the removed testicle was 5.9 mL.

### 3.3 History of cryptorchidism

Twenty‐five of 334 men (7.5%) had a history of cryptorchidism as their only risk factor. Biopsies were offered to 22 of these and 19 accepted. No cases of GCNIS were detected in the group.

### 3.4 Unknown fertility status

**FIGURE 1:**
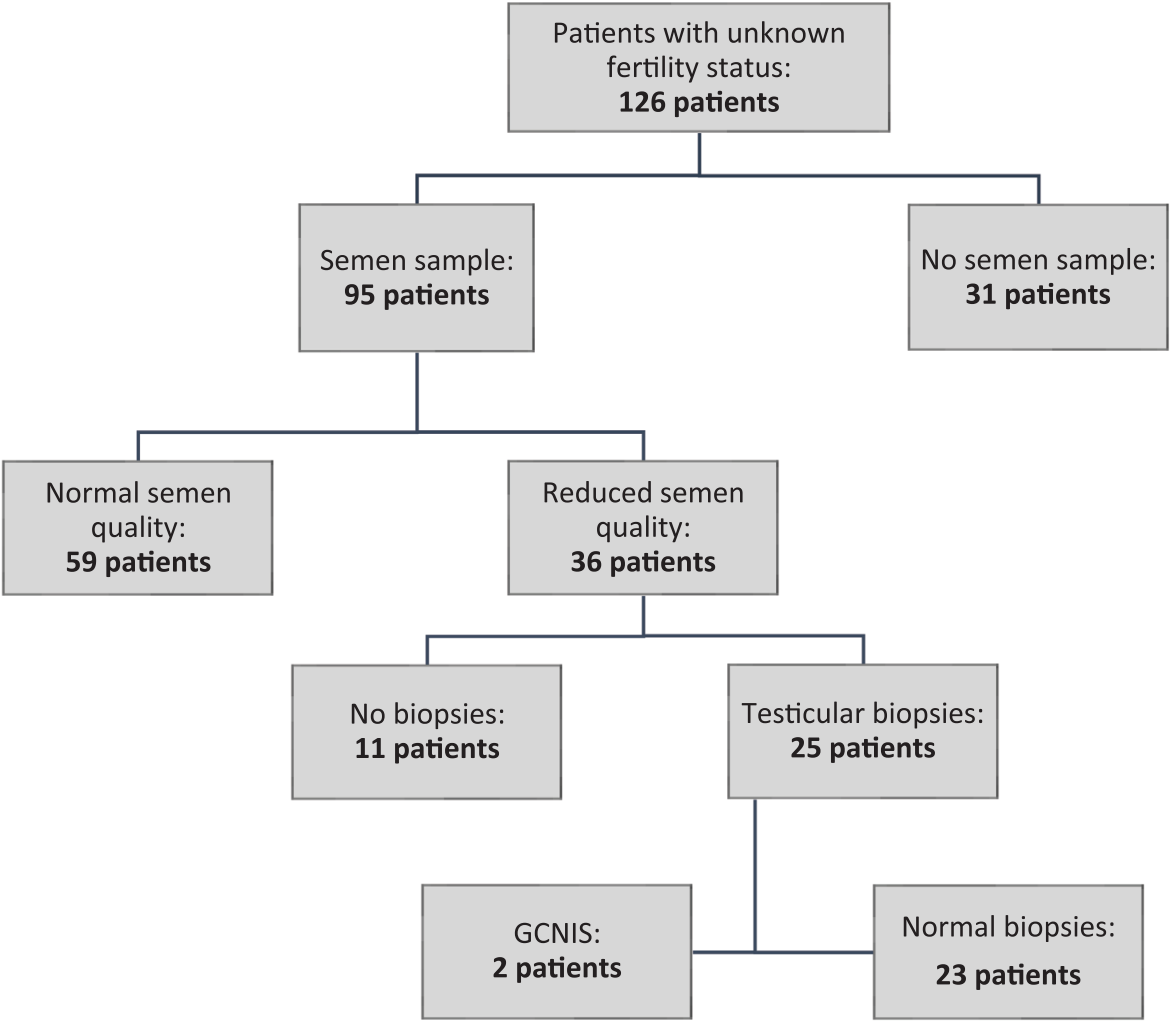
Flowchart of the trajectory for men with testicular microlithiasis and unknown fertility status. GCNIS, germ cell neoplasia in situ.

Overall, 126 of 334 men (37.7%) had unknown fertility status and no other risk factors. Ninety‐five men with TM and unknown fertility status underwent semen analyses. Out of the 31 cases where semen samples were not collected, four were because of patient age, 11 were because of patient refusal or absence from appointments, in 12 cases the samples were not offered by the treating physician, and in four cases the patients were referred directly to biopsies (see below). Thirty‐six of the 95 patients (38%) who underwent semen analyses had reduced semen quality. Of them, 25 underwent biopsies. For the 11 men who did not undergo biopsies reasons included patient refusal or absence from appointments (*n* = 5), previous biopsies without GCNIS (*n* = 1), deferral because of desired pregnancy (*n* = 1), age >50 years (*n* = 1), and clinical decision based on semen parameters being only marginally reduced (*n* = 3). GCNIS was detected in two of the biopsies corresponding to 8% (95% CI 0.01%–26%) of the 25 biopsied men. On the pathology there were no incidents of cancer, and the removed testicles were 7.3 and 12 mL, respectively. Figure 1 depicts the trajectory for patients with TM and unknown fertility status.

### 3.5 Multiple risk factors

Overall, 56 of 334 men (16.8%) had multiple risk factors. Fifty‐two of these were offered biopsies, while follow‐up without biopsies were offered in four cases because of fertility concerns. Forty‐seven of 52 men (90.4%) accepted the offer. GCNIS was detected in four of 47 men (8.5%, 95% CI 2.4%–20.4%). The risk factors among these patients included testicular hypotrophy and infertility in two cases, and testicular hypotrophy, infertility, and a history of cryptorchidism in two other cases. On orchidectomy, early‐stage cancer was present in three of four of the GCNIS carrying testicles.

### 3.6 No risk factors

Biopsies were offered and performed on 17 men outside of the standard recommendations: four men with unknown fertility status who lacked semen analyses, six men with normal semen parameters according to WHO criteria, and seven men with no risk factors for testicular cancer who had previously fathered children. No cases of GCNIS were detected in this group.

### 3.7 Complications

Complications to the biopsies were noted in 25 of 137 men (18.2%, 95% CI 11.9%–24.9%). These included bleeding (*n* = 2), post‐operative pain (*n* = 13), minor wound defects (*n* = 3), epididymal damage (*n* = 2), wound infection (*n* = 4), and hydrocele (*n* = 1). Most cases were handled conservatively corresponding to Clavien‐Dindo grade 1 complications. Still, oral antibiotics were given for the wound infections and one patient underwent surgery for his hydrocele, corresponding to Clavien‐Dindo grades 2 and 3a, respectively.

## 4 DISCUSSION

We followed a large cohort of men with incidentally detected TM diagnosed over a 10‐year period in Denmark. The overall incidence rate of GCNIS was 3%, and with up to 11 years of follow‐up, an additional 0.9% of the cohort developed testicular cancer. Notably, negative GCNIS biopsies did not rule out subsequent cancer development. GCNIS was exclusively found in testicles with TM and was associated with a testicular size of less than 12 mL in nine of 10 of cases. Importantly, overlapping data from our previous study^8^ showed that two patients with TM and a history of infertility and/or reduced semen quality were diagnosed with GCNIS. A more thorough review of pathology reports in the present study revealed that both patients also had reduced testicular size (<12 mL).

Since GCNIS will develop into testicular cancer in most cases, its early detection is desired.^12^ Men with TM represents a group of interest in this regard. European and Danish urological guidelines recommend testicular biopsies for men younger than 50 years with TM and additional risk factors for testicular cancer as described above.^6^, ^13^ Moreover, the European guidelines recommend biopsies in men with bilateral TM, while the Danish advocate for semen analyses and potential biopsies in men with unknown fertility status.

Our group has recently questioned the association between TM and GCNIS for men with normal testicular size in a study by Frandsen et al.^8^ The case series of 167 biopsied men with TM showed a statistically significantly increased risk of GCNIS in men with testicular hypotrophy with an odds ratio of 9.36 (95% CI: 2.41, 61.88; *p *= 0.004), while no patients with previous cryptorchidism and normal testicular size were found to harbor GCNIS.^8^ As described above, the additional GCNIS cases in two subfertile men were also associated with testicular hypotrophy. This means that the two studies represent a period of 16 years (2007‒2023) in eastern Denmark with no finding of GCNIS in any patients with TM and normal testicular size.

The current guidelines^6^ base their recommendations regarding biopsies in infertile men largely on a systematic review and meta‐analysis of eight case‒control studies, with a total of 180 infertile men with TM. This review concluded that there was an approximately 18‐fold higher odds ratio for GCNIS or testicular cancer in infertile men with TM than men without TM.^14^ However, when considering the included studies individually, several major issues are apparent: a study by La Vignera et al. carried the greatest weight in the pooled meta‐analysis, despite significant limitations. It did not include data on testicular volume or medical history, and only 67% of the TM group (60 men) exhibited reduced semen quality.^15^ The study documented testicular tumors in 10 of the infertile men, but did not determine whether these men had any additional risk factors. In another of the eight studies, the authors evaluated 2172 men from infertile couples and found TM in 31 of these.^16^ Overall, 11 cases of GCNIS or germ cell tumors were identified through clinical examination, biopsies, or US follow‐up over a period of up to 9 years. Among these, four cases were associated with TM. However, three of these four cases also presented with hypotrophy in one or both testicles, characterized by a volume of less than 12 mL. No instances of GCNIS or testicular cancer were found in the remaining six studies.^17^, ^18^, ^19^, ^20^, ^21^, ^22^ However, in the meta‐analysis, two patients with TM were classified as cancer cases because of a prior diagnosis and orchiectomies performed before the studies. In a subsequent retrospective cross‐sectional study, also referenced in the European Urological guidelines.^6^ De Gouveia Brazao et al. examined 263 infertile men of whom 53 had TM.^23^ Biopsies showed GCNIS in six of the 53 men, all with bilateral TM. Five of the men with GCNIS also had a history of cryptorchidism and four of these had a decreased testicular size in the GCNIS‐carrying testicle compared with the contralateral testicle. The sixth person with GCNIS had no cryptorchidism and normal testis size. Thus, three out of 233 infertile men with TM were diagnosed with GCNIS or developed germ cell tumors, despite having normal testicular size. This corresponds to an incidence rate of 1.3%, which aligns with what would be expected in infertile men irrespective of the presence of TM.

In relation to cryptorchidism the literature is quite sparse. In 2005, Husmann noted that two of 19 patients with TM who underwent orchiopexy because of cryptorchidism, developed testicular cancer over a median follow‐up of 8 years.^24^ Meanwhile, patients' age and information on additional risk factors, including testicular hypotrophy, was omitted and no control group without TM was described. Another study followed 163 males with TM after primary orchiopexy and detected no cancer development, with a median follow‐up of 6.3 years.^25^ Thus, there is very limited evidence for an elevated risk in men with a combination of TM and cryptorchidism without testicular hypotrophy. The notion in the Guidelines of the European Urological Association that biopsies in men with bilateral TM are indicated in men with no other risk factors seem to be undocumented as no original study has described this.^6^ In contrast, several studies have uniformly shown that TM is not associated with cancer development in otherwise healthy men.^2^, ^3^, ^26^, ^27^, ^28^, ^29^, ^30^ Finally, there is insufficient data, both in our and previous studies, to make conclusions on men with TM and a family history of testicular cancer. With our findings and the results from previous studies in mind, there is a considerable chance that current urological guideline recommendations lead to overtreatment of men with TM.

Another unique scenario involves TM in the remaining testis of men treated for testicular cancer. These individuals were not included in our study, as contralateral biopsies have been routinely performed in Denmark in cases of testicular cancer to rule out concomitant GCNIS. However, previous reports indicate that the presence of TM in the contralateral testis is associated with an increased risk of GCNIS in these biopsies.^31^ Additionally, a recent study has shown an elevated risk of subsequent cancer development during long‐term follow‐up in non‐biopsied contralateral testicles of cancer patients.^32^ Therefore, performing biopsies in this group appears to be a reasonable approach. Periodic US may be considered as an alternative to biopsies for patients likely to adhere to regular monitoring, taking into account concerns related to cost and potential anxiety. However, US as a general strategy may not be ideal as the compliance with such programs has been shown to be limited.^33^

Our study represents the largest case series on patients with TM to date. However, the retrospective nature of the study inherently limits causality and introduces potential selection bias. Specifically, we relied on the documentation available in patient charts, which may have led to inconsistencies in data collection, especially regarding testicular assessments, which were often based on clinical evaluations rather than standardized imaging measurements. We could only confirm testicular size from pathology reports in cases where patients underwent orchidectomy. This may have led to an underestimation of the true incidence of testicular hypotrophy in our cohort and therefore overestimation of the risk associated with hypotrophy. Likewise, not all men with unknown fertility status underwent semen analyses which may have led to an underestimation of the proportion of men with reduced semen quality. Furthermore, the relatively short follow‐up period for some patients may suggest that a larger proportion of the cohort could develop testicular cancer in the future. As an additional limitation, the study cohort consisted solely of men residing in Denmark from the same geographic region thereby limiting the study's external validity. Finally, the variability in follow‐up duration should be noted. Given the long latency period for GCNIS progression to testicular cancer, shorter follow‐up times may underestimate cancer incidence. Studies with longer follow‐up would enhance the reliability of our findings.

## 5 CONCLUSIONS

Although the level of evidence is generally limited, both our findings and previous studies suggest that biopsies should only be considered in men with incidental testicular microlithiasis and no other ultrasonic signs indicative of malignancy if the testicular size is reduced. The cut‐off of 12 mL seems clinically relevant in this regard although a precise recommendation is difficult to make. As subsequent testicular cancers may develop regardless of biopsy status, it is reasonable to advise regular self‐palpation in all patients.

## AUTHOR CONTRIBUTIONS

*Conceptualization*: Rasmus Hassing Frandsen, Christian Fuglesang S. Jensen, Peter Busch Østergren, Jens Sønksen, and Mikkel Fode. *Methodology*: Christian Fuglesang S. Jensen, Peter Busch Østergren, Jens Sønksen, and Mikkel Fode. *Investigation*: Karoline Skov Lundager, Rasmus Hassing Frandsen, Emil Durukan, and Nadia Zeeberg Belhouche. *Data curation*: Mikkel Fode. *Formal analysis*: Mikkel Fode. *Writing—original draft*: Karoline Skov Lundager and Mikkel Fode. *Writing—review and editing*: Karoline Skov Lundager, Rasmus Hassing Frandsen, Emil Durukan, Nadia Zeeberg Belhouche, Christian Fuglesang S. Jensen, Peter Busch Østergren, Jens Sønksen, and Mikkel Fode. *Project administration*: Mikkel Fode.

## CONFLICT OF INTEREST STATEMENT

The authors declare they have no conflicts of interest.

## FUNDING INFORMATION

The authors received no specific funding for this work.

## Data Availability

The data that support the findings of this study are available from the corresponding author upon reasonable request. Data sharing is subject to approval from the Regional Center for Register Research of the Capital Region of Denmark.
